# Hydroa vacciniforme-like lymphoproliferative disorder in Korea

**DOI:** 10.1038/s41598-020-76345-2

**Published:** 2020-11-09

**Authors:** Byeol Han, Keunyoung Hur, Jungyoon Ohn, Tae Min Kim, Yoon Kyung Jeon, You Chan Kim, Je-Ho Mun

**Affiliations:** 1 Department of Dermatology, Seoul National University College of Medicine, Seoul, Republic of Korea; 2 Institute of Human–Environment Interface Biology, Seoul National University, Seoul, Republic of Korea; 3 Department of Internal Medicine, Seoul National University Hospital, Seoul, Republic of Korea; 4 Seoul National University Cancer Research Institute, Seoul, Republic of Korea; 5 Department of Pathology, Seoul National University College of Medicine, Seoul, Republic of Korea; 6 Department of Dermatology, Ajou University School of Medicine, Suwon, Republic of Korea

**Keywords:** Haematological cancer, Cancer, Haematological diseases, Skin diseases, Oncology

## Abstract

Hydroa vacciniforme-like lymphoproliferative disorder (HVLPD) is a rare Epstein–Barr virus (EBV)-associated lymphoproliferative disease. The disease course of HVLPD varies from an indolent course to progression to aggressive lymphoma. We investigated the characteristics of HVLPD in Korean patients. HVLPD patients at Seoul National University Hospital between 1988 and 2019 were retrospectively analyzed. This study included 26 HVLPD patients who all presented with recurrent papulovesicular and necrotic eruption on the face, neck, and extremities. EBV was detected from the skin tissues of all patients. HVLPD was diagnosed during childhood (age < 18 years) in seven patients (26.9%) and in adulthood (age ≥ 18 years) in 19 cases (73.1%). The median age at diagnosis was 24.0 years (range 7–70 years). HVLPD has various clinical courses, from an indolent course to progression to systemic lymphoma. Fourteen patients (53.8%) developed lymphoma: systemic EBV-positive T-cell lymphoma (n = 9, 34.6%); extranodal natural killer/T-cell lymphoma, nasal type (n = 3, 11.5%); aggressive natural killer/T-cell leukemia (n = 1, 3.8%); and EBV-positive Hodgkin lymphoma (n = 1, 3.8%). Mortality due to HVLPD occurred in five patients (26.3%) in the adult group, while it was one patient (14.3%) in the child group. As lymphoma progression and mortality occur not only in childhood but also in adulthood, adult-onset cases may need more careful monitoring.

## Introduction

Hydroa vacciniforme-like lymphoproliferative disorder (HVLPD) is a rare Epstein–Barr virus (EBV)-associated T-cell or natural killer (NK)-cell lymphoproliferative disease. According to the revised 4th World Health Organization (WHO) classification, the term HVLPD has been renamed from hydroa vacciniforme-like lymphoma to HVLPD owing to its relationship to chronic active EBV infection (CAEBV) and the broad spectrum of its clinical course^1^. Although HVLPD usually affects children, adult and elderly cases have been reported^2^. Clinically, it is characterized by recurrent erythematous papules and vesicles that leave pitted scars on sun-exposed areas, including the face and forearms. The disease course of HVLPD varies from an indolent course to systemic lymphoma. Our experience and the literature show that mortality or a fatal outcome from the progression of HVLPD to lymphoma can also occur in adults^3–6^. In the present study, we report on 26 cases of HVLPD encountered at our hospital during the last three decades.

## Results

### Clinical features

**Figure 1:**
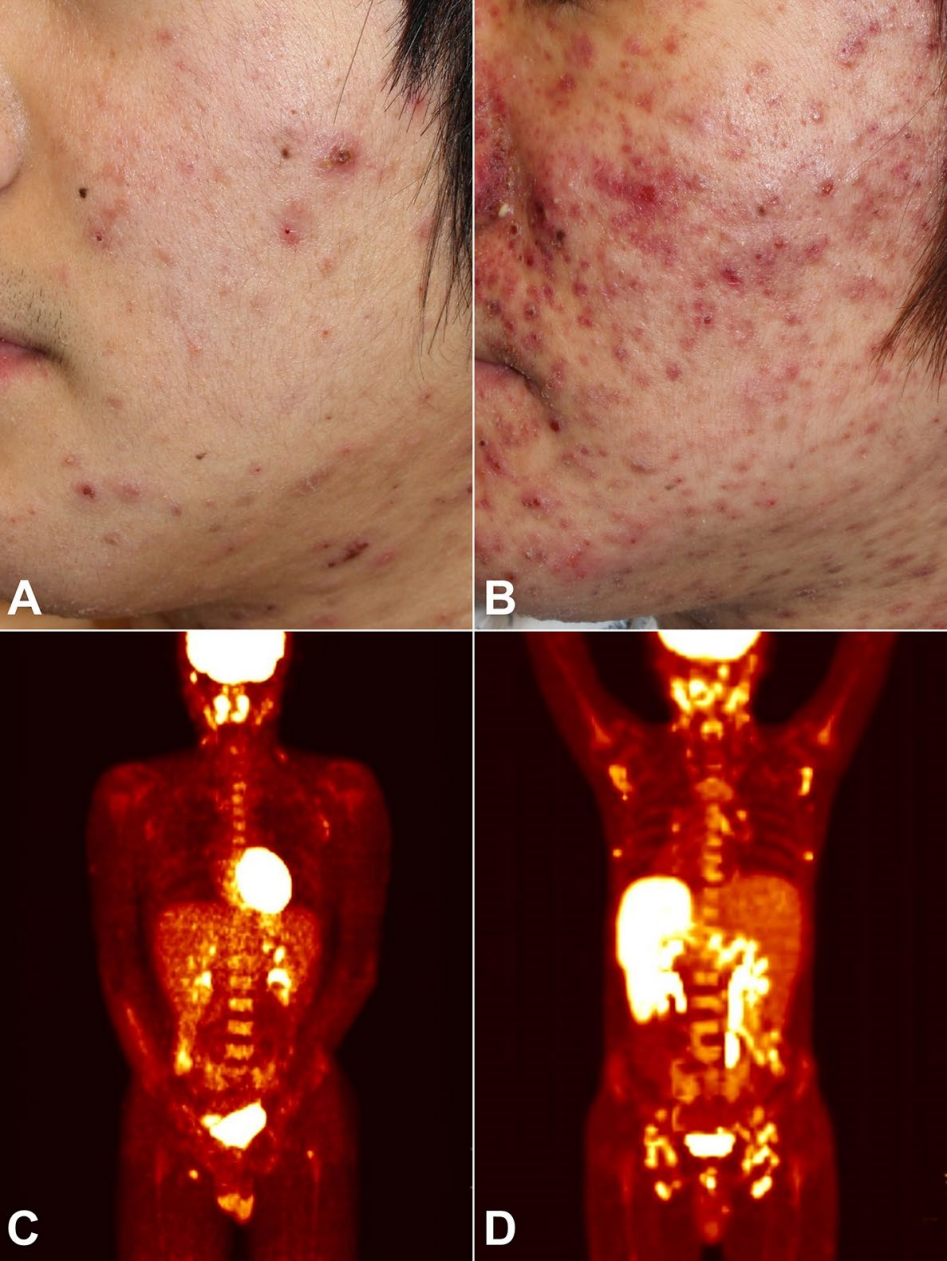
An 18-year-old adult (patient no. 8) diagnosed with hydroa vacciniforme-like lymphoproliferative disease that progressed to systemic Epstein-Barr virus-positive (EBV +) T-cell lymphoma. (**A**) Erythematous crusted necrotic papulovesicles on the face. (**B**) Six months after the diagnosis, the skin lesions became aggravated. At this stage, systemic EBV+ T-cell lymphoma was diagnosed. (**C**) In the positron emission tomography-computed tomography (PET-CT) scan, there was no evidence of systemic involvement at the first visit. (**D**) However, as the skin eruption deteriorated (**B**), the PET-CT scan revealed multiple hypermetabolic lesions in the cervical, axillary, abdominal, and inguinal lymph nodes and in the spleen.

A total of 26 cases were enrolled with diagnosis of HVLPD based on clinical and histopathologic features between 1988 and 2019 in the Department of Dermatology of Seoul National University Hospital (SNUH). Fifteen cases had been previously reported^7–13^. There were seven children (age < 18 years) and 19 adults (age ≥ 18 years), with a median age of 24.0 years (range 7–70 years) at the time of diagnosis. The age at which the skin lesions of HVLPD first appeared varied, with a median age of 17.5 years (range 3–70 years). Among 19 adult patients, 6 (31.6%) reported that they had skin lesions in childhood. There were 16 male patients and 10 female patients (male-to-female ratio 1.6:1). All patients had recurrent vesicles, papules, crusted papulovesicles, necrotic ulcers, and pitted scars on sun-exposed areas (Fig. 1 and Tables 1 and 2). Besides lesions on the face and arms, skin lesions on non-sun-exposed areas, including the trunk, were present in seven patients (26.9%). Oral ulcer was noted in nine patients (34.6%), and facial swelling was observed in three patients (11.5%). At the first consultation, six patients (23.1%) had fever and cervical lymphadenopathy and two patients (7.7%) had splenomegaly. The median follow-up duration was 36.0 months (range 12–216 months). Ultraviolet (UV) A radiation provocation test was performed in seven patients. Among them, five patients (71.4%) had skin eruption with papulovesicles. Mosquito bite hypersensitivity was noted in three patients (11.5%). Serum EBV viral load was evaluated in 11 patients (42.3%). All patients showed serum EBV. High levels of viral load, ranging from 54,275 to 47,925,563 copies/mL (median 1,784,901 copies/mL), were observed during follow-up.

**Table 1:** Summary of characteristics of patients with hydroa vacciniforme-like lymphoproliferative disease.

Variable	Total N = 26, n (%)
**Age at diagnosis, mean ± SD (range) (years)**	25.3 ± 12.8 (7–70)
Children (< 18)	7 (26.9)
Adult (≥ 18)	19 (73.1)
**Age at onset, mean ± SD (range) (years)**	19.8 ± 14.9 (3–70)
During childhood (< 18)	13 (50.0)
During adulthood (≥ 18)	13 (50.0)
**Disease duration, mean ± SD (range) (years)**	5.5 ± 5.2 (0–17)
**Sex**	
Male	16 (61.5)
Female	10 (38.5)
**Anatomic location of cutaneous manifestations**	
Sun-exposed area	26 (100)
Non–sun-exposed area	7 (26.9)
**Extracutaneous involvement**	
Yes	16 (61.5)
No	10 (38.5)
**UVA provocation test [n = 7]**^a^	
Positive	5 (71.4)
Negative	2 (28.6)
**Serum EBV DNA load, mean ± SD (range) (copies/mL) [n = 11]**^b^	9,849,887.6 ± 15,824,779.4 (54,275–47,925,563)
**Development of systemic lymphoma**	
Yes	14 (53.8)
Systemic EBV+ T-cell lymphoma	9 (34.6)
Extranodal NK/T-cell lymphoma, nasal type	3 (11.5)
Aggressive NK/T-cell leukemia	1 (3.8)
EBV+ Hodgkin lymphoma	1 (3.8)
No	12 (46.2)
**Outcome**	
Alive with disease	19 (73.1)
Mortality due to the disease	6 (23.1)
Mortality due to another disease	1 (3.8)

*EBV+* Epstein–Barr Virus-positive, *NK* natural killer, *SD* standard deviation, *UVA* ultraviolet A.

^a^UVA provocation test was performed in seven patients.

^b^Serum EBV DNA load was evaluated in 11 patients.

**Table 2:** Clinical features and histopathologic findings in patients with hydroa vacciniforme-like lymphoproliferative disease at our institution.

Patient no.	Sex	Age at diagnosis, years (age at onset, years)	Cutaneous manifestations	IHC, EBER-1 ISH, TCRγ gene rearrangement	Serum EBV DNA load (copies/mL)	UVA provocation test	Extracutaneous involvement	Treatment	Disease course	Follow-up (months)	Last status during follow-up
1	F	7 (6)	Erythematous crusted papulovesicles on the face and oral ulcer	CD3+, CD20+ a few, Ki-67 10%, EBV ISH+	15,462,763	Positive	None	Observation	Partial remission and recurrence	72	AWD
2	M	10 (9)	Erythematous crusted papulovesicles on the face	CD3+, CD20+ a few, CD56−, EBV ISH+	ND	Negative	None	Systemic steroid, minocycline	Partial remission and recurrence	216	AWD
3^7^	F	11 (5)	Erythematous crusted papulovesicles on the face and arm, and oral ulcer	CD3+, CD45RO+, CD8+, CD20−, CD56+ few, LMP-1−, EBV ISH+	1,784,901	ND	None	Systemic steroid on recurrence	Partial remission and recurrence	180	AWD
4^8^	F	12 (3)	Erythematous crusted papulovesicles on the face, arm, and leg	CD3+, CD45RO+, CD56−, S-100−, lysozyme+ focal, EBV ISH+	ND	ND	Liver, spleen, bone marrow	Oral acyclovir	Progressed to aggressive NK-cell leukemia	108	DOD
5^9^	F	15 (10)	Erythematous crusted papulovesicles on the face and arm	CD3+, CD45RO+, CD20+ a few, CD56−, Ki-67+ a few, LMP-1−, EBV ISH+, TCR gamma gene arrangement−	ND	Negative	None	Observation	Remission	36	AWD
6	M	17 (17)	Erythematous crusted papulovesicles on the face and trunk, and oral ulcer	CD3+, CD4+, CD8+ focal, CD20−, CD56−, TIA-1+, Ki-67 70%, EBV ISH+, TCR gamma gene rearrangement−	8,463,700	ND	Bone marrow	Systemic steroid → chemotherapy	Progressed to systemic EBV+ T-cell lymphoma	12	AWD
7^10^	M	17 (8)	Erythematous crusted papulovesicles on the face and forearm	CD45RO+, CD20−, EBV ISH+	ND	Negative	Cervical lymph node, liver, spleen	Systemic steroid, chemotherapy	Progressed to systemic EBV+ T-cell lymphoma	NS	AWD
8	M	18 (6)	Erythematous crusted papulovesicles on the face, arm, and trunk	CD3+, CD4+, CD8+ focal, CD20−, CD30+ a few, CD56+, granzyme B+, Ki-67 10%, EBV ISH+	47,925,563	Positive	Cervical lymph node, spleen, iliac bone, bone marrow	Systemic steroid → chemotherapy	Progressed to systemic EBV+ T-cell lymphoma	12	DOD
9	F	19 (17)	Erythematous crusted papulovesicles on the face and arm, oral ulcer, and facial swelling	CD3+, CD4−, CD8−, CD20−, CD56−, CD30−, EBV ISH+	693,642	ND	right breast, lung, spleen, liver, pelvic bone, bone marrow	Systemic steroid → chemotherapy	Progressed to extranodal NK/T-cell lymphoma, nasal type	24	DOD
10	M	19 (4)	Erythematous crusted papulovesicles on the face, arm, trunk, and ankle	CD20+ a few, CD56+ a few, LMP-1−, EBV ISH+	79,396	ND	None	Observation	Remission	204	AWD
11^10^	M	19 (4)	Erythematous crusted papulovesicles on the face and forearm	CD45RO+, CD20−, EBV ISH+	ND	Negative	None	Systemic steroid, oral acyclovir	Partial remission	NS	AWD
12^10^	M	21 (19)	Erythematous crusted papulovesicles on the face and oral ulcer	CD45RO+, CD20−, EBV ISH+	ND	Negative	Cervical lymph node, liver	Systemic steroid	Progressed to Systemic EBV+ T-cell lymphoma	NS	DOD
13	M	24 (10)	Erythematous crusted papulovesicles on the face, arm, and trunk	CD3+, CD45RO+, CD4+, CD8+, CD20+ a few, CD56−, granzyme B+ a few, Ki-67 1%, EBV ISH+, TCR gamma gene rearrangement−	1,840,744	ND	Liver, spleen, bone marrow	Systemic steroid → chemotherapy	Progressed to Systemic EBV+ T-cell lymphoma	120	DOD
14^10^	M	24 (18)	Erythematous crusted papulovesicles on the face and forearm	CD45RO+, CD20−, EBV ISH+	ND	Negative	Cervical lymph node, spleen	Systemic steroid	Progressed to peripheral T-cell lymphoma of adulthood	NS	DOD
15	M	26 (25)	Erythematous crusted papulovesicles on the face	CD3+, CD4+, CD8+ focal, CD 20+ a few, CD30+ focal, CD56−, Ki-67 15%, TCR gamma gene rearrangement+	30,917,751	ND	Cervical and axillary lymph node, spleen	Systemic steroid → chemotherapy	Progressed to peripheral T-cell lymphoma of adulthood	204	AWD
16^9^	M	28 (22)	Erythematous crusted papulovesicles on the face	CD3+, CD45RO+, CD20+ a few, CD56−, Ki-67+ a few, LMP-1−, EBV ISH+, TCR gene rearrangement−	ND	Positive	None	Observation	Remission	36	AWD
17^9^	F	28 (26)	Erythematous crusted papulovesicles on the face and facial swelling	CD3+, CD45RO+, CD20+ a few, CD56−, Ki-67 15%, EBV ISH+, TCR gamma gene rearrangement−	ND	ND	None	Systemic steroid	Partial remission and recurrence	36	Death
18^9^	M	29 (25)	Erythematous crusted papulovesicles on the face, arm, and trunk	CD3+, CD45RO+, CD20+ a few, CD56−, Ki-67+ (< 5%), EBV ISH+, TCR gamma gene rearrangement−	ND	Positive	None	Systemic steroid	Partial remission and recurrence	36	AWD
19	F	30 (27)	Erythematous crusted papulovesicles on the face and oral ulcer	CD3+, CD45RO+, CD20+ a few, CD56−, LMP-1−, Ki-67 < 5%, EBV ISH+, TCR gamma gene rearrangement+	ND	ND	Cervical and axillary lymph node	Chemotherapy	Progressed to or accompanied extranodal NK/T-cell lymphoma, nasal type	12	AWD
20	M	32 (15)	Erythematous crusted papulovesicles on the face and oral ulcer	CD3+, CD20+ a few, CD56−, granzyme B+, EBV ISH+, Ki-67 20%	54,275	ND	Palate, bone marrow	Chemotherapy, daratumumab	Progressed to extranodal NK/T-cell lymphoma, nasal type	48	AWD
21^11^	F	33 (30)	Erythematous crusted papulovesicles on the face	CD3+, CD45RO+, CD20−, CD30−, CD56−, Ki-67 20%, EBV ISH+, TCR gamma gene rearrangement−	ND	Positive	Cervical lymph node, stomach	Chemotherapy	Progressed to Systemic EBV+ T-cell lymphoma	18	AWD
22^11^	M	34 (33)	Erythematous crusted papulovesicles on the face, arm, and trunk, and oral ulcer	CD3+, CD45RO+, CD20−, CD30−, CD56−, Ki-67 20%, EBV ISH+, TCR gamma gene rearrangement+	ND	ND	Bone marrow	Chemotherapy	Progressed to Systemic EBV+ T-cell lymphoma	24	AWD
23^9^	M	37 (32)	Erythematous crusted papulovesicles on the face, arm, and trunk	CD3+, CD45RO+, CD20+ a few, CD56−, Ki-67+ (< 5%), EBV ISH+, TCR gamma gene rearrangement−	ND	ND	Cervical lymph node	Chemotherapy	Partial remission and recurrence	36	AWD
24	M	38 (38)	Erythematous crusted papulovesicles on the face	CD3+, CD4+ focal, CD8+ focal, CD20−, CD56−, granzyme B+ focal, Ki-67+, EBV ISH+, TCR gamma gene rearrangement+	925,529	ND	Lung (Hodgkin lymphoma), bone marrow, spleen, bone	Systemic steroid → chemotherapy	Progressed to EBV+ Hodgkin lymphoma	96	AWD
25^9^	F	39 (36)	Erythematous crusted papulovesicles on the face, arm, and trunk	CD3+, CD45RO+, CD20+ a few, CD56−, Ki-67+ (< 5%), EBV ISH+, TCR gamma gene rearrangement−	ND	Positive	Cervical lymph node	Chemotherapy	Partial remission and recurrence	36	AWD
26^12^	F	70 (70)	Erythematous crusted papulovesicles on the face, oral ulcer, and facial swelling	CD2+, CD3+, CD4+ a few, CD5+, CD8+ focal, CD20−, CD30−, CD56−, EBV ISH	200,500	Negative	None	Topical steroid	Partial remission and recurrence	12	AWD

*AWD* alive with the disease, *DOD* died of disease, *EBER-1 ISH* EBV-encoded small nonpolyadenylated RNA (EBER)-1 by in situ hybridization, *F* female, *IHC* immunohistochemistry, *M* male, *ND* not done, *NS* not specified exact follow-up duration.

### Histopathologic features, immunohistochemical findings, in situ hybridization, and molecular studies

**Figure 2:**
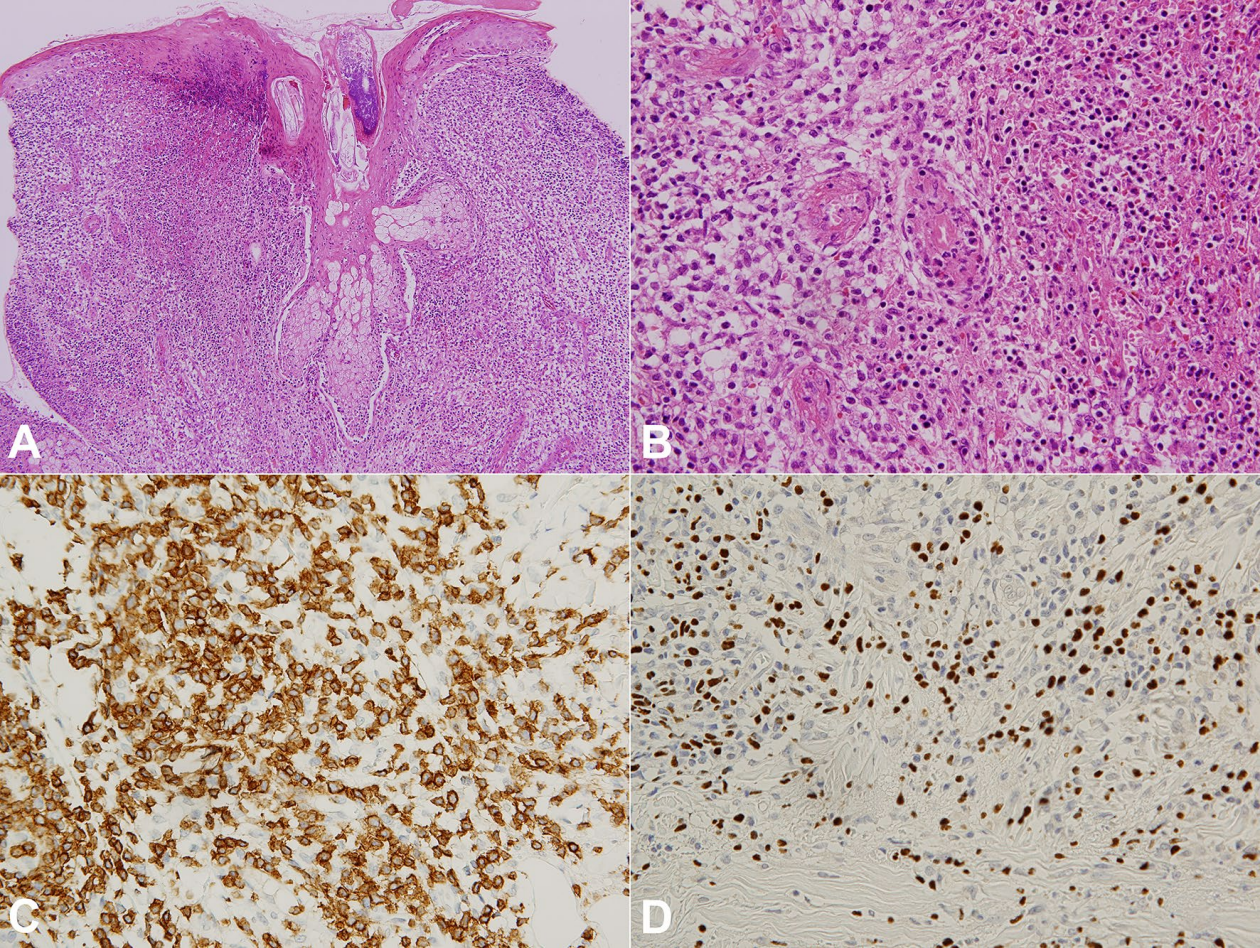
Histopathologic findings of hydroa vacciniforme-like lymphoproliferative disease. (**A**) Histopathologic analysis showed diffuse and dense infiltration of lymphocytes in the dermis. Epidermal necrosis, subepidermal edema, and perivascular and periappendageal atypical lymphocytic infiltration were observed (hematoxylin and eosin staining ×100). (**B**) Small- to medium-sized atypical lymphocytes with nuclear hyperchromasia were detected (hematoxylin and eosin staining ×400). (**C**) The atypical mononuclear cells were positive for CD3 in immunohistochemical staining (×400). (**D**) In situ hybridization for EBV-encoded small nonpolyadenylated RNA was positive in the atypical mononuclear cells (×400).

The histopathologic features of cutaneous tissue were similar in all patients. Dense and diffuse infiltration of atypical lymphocytes was seen in the dermis and subcutaneous fat (Fig. 2). Epidermal necrosis, vesiculation, subepidermal edema, and perivascular and periappendageal atypical lymphocytic infiltration were found. The proliferated atypical lymphocytes were of small to intermediate size, with enlarged, round to oval nuclei. There were an inflammatory background with lymphocytes, eosinophils, histiocytes and plasma cells. The immunohistochemistry results and molecular study findings are summarized in Table 2. EBV-encoded small nonpolyadenylated RNA (EBER) was positive in all patients. EBER-positive cells were predominantly seen in the perivascular and periappendageal area in the dermis and subcutaneous fat. Detection of monoclonal T-cell receptor γ gene rearrangements was performed in 13 patients. Among them, four patients (30.8%) showed these rearrangements.

### Treatment and disease course

Various treatments were applied. Sun exposure avoidance was recommended to all patients. Systemic steroid was used to control the disease in 14 patients (53.8%). Systemic acyclovir or minocycline was administered in three patients. Thirteen patients received chemotherapy as the first-line or second-line therapy because of disease activity exacerbation. Seven patients died during the follow-up (mortality rate 26.9%), six of whom died of the disease; one of whom (case no. 17) died of breast cancer. Therefore, HVLPD mortality occurred in seven patients (23.1%).

In the child group, one patient showed remission (14.3%) and three patients had partial remission and recurrence (42.9%). The disease progressed to systemic lymphoma or leukemia in three patients (42.9%), two of whom had a diagnosis of systemic EBV-positive (EBV+) T-cell lymphoma of childhood and one of whom had aggressive NK/T-cell leukemia. The mortality rate of HVLPD in the child group was 14.3% (1/7). The cause of death was aggressive NK/T-cell leukemia.

In the adult group, remission was noted in two patients (10.5%) and partial remission was observed in six patients (31.6%). Eleven cases (57.9%) were associated with systemic lymphoma, of which seven (36.8%) progressed to systemic EBV+ T-cell lymphoma; 3 (15.8%) to extranodal NK/T-cell lymphoma, nasal type; and 1 (5.3%) to EBV+ Hodgkin lymphoma. The mortality rate of HVLPD in the adult group was 31.6% (6 of 19). When we excluded the case of breast cancer death, the rate of disease-specific death was 26.3% (5 of 19) in the adult group. The causes of death were progression to systemic EBV+ T-cell lymphoma in four patients and extranodal NK/T-cell lymphoma, nasal type, in one patient.

### Progression to lymphoma and mortality in relation to age at diagnosis, disease onset, and disease duration

We compared the rates of HVLPD progression to lymphoma and disease-related mortality between patients diagnosed during childhood and those diagnosed during adulthood. Although the rates of both were higher among those diagnosed during adulthood, the differences were not statistically significant (p = 0.665 for progression and p = 1.000 for mortality). We further analyzed these parameters according to whether disease onset occurred in childhood (age < 18 years) or adulthood (age ≥ 18 years). There was no difference between the two groups with regard to either progression to lymphoma or disease-related mortality (p = 1.000 and p = 0.645, respectively). Additionally, we evaluated the effect of the period from disease onset to first diagnosis, comparing patients with a disease duration of < 10 years and those with a duration of ≥ 10 years. There were no differences in the rate of progression to lymphoma or disease-related mortality (p = 1.000 and p = 0.218, respectively) in relation to disease duration before first diagnosis.

## Discussion

Hydroa vacciniforme is a rare photodermatosis originally described by Bazin^14^. Although the pathogenesis of hydroa vacciniforme is not clearly established, sensitivity to UVB or UVA has been suggested as a pathologic mechanism^15^. In 1996, Cho et al. first suggested an association with EBV and recurrent necrotic papulovesicular eruptions of the face^10^. As most cases with hydroa vacciniforme manifestations have shown the presence of EBV, the question about the existence of classic hydroa vacciniforme without EBV infection remains unresolved^16^. Therefore, Quintanilla-Martinez and Fend suggested renaming the disease as hydroa vacciniforme EBV-associated lymphoproliferative disorder to encompass the broad clinical spectrum^16^.

HVLPD has been given various names, including hydroa vacciniforme-like lymphoma and EBV-associated vesiculopapular eruption on the face. In 2008, the WHO classification introduced 2 T-cell lymphoproliferative disorders associated with EBV in children: hydroa vacciniforme-like lymphoma and systemic EBV+ lymphoproliferative disease of childhood^17^. In 2016, the 4th WHO classification reclassified the disease into two groups: HVLPD and systemic EBV+ T-cell lymphoma of childhood (Table 3)^18^.

**Table 3:** Change of definition of hydroa vacciniforme and hydroa vacciniforme-like lymphoproliferative disease.

Diagnosis	Definition	References
Hydroa vacciniforme	Rare, sporadic, idiopathic photodermatosis characterized by papules, vesicles, and crusts which heal with vacciniform scarring after sunlight exposure. The pathogenesis is unclear although sensitivity of UVB or UVA has been suggested	First reported by Bazin^14,15^
Hydroa vacciniforme-like lymphoma	Proliferation of clonal T cells or, less frequently, NK cells infected by EBV with a latency type 1 profile. It has an indolent clinical course with long periods of recurrent skin lesions in sun-exposed areas that tends to regress spontaneously. After several years, the process may resolve or progress to systemic disease	2008 WHO classification^17^
Systemic EBV+ lymphoproliferative disease of childhood	Aggressive condition with rapid evolution to multiple-organ failure and death. It has overlapping features with aggressive NK-cell leukemia, but the cells have a T-cell phenotype and clonal TCR rearrangement. It may emerge in a background of chronic EBV infection and progress from a polyclonal, to an oligoclonal, and to a monoclonal EBV-driven proliferation	
Hydroa vacciniforme-like lymphoproliferative disease	Name changed from lymphoma to lymphoproliferative disorder due to its relationship with chronic active EBV infection and the broad spectrum of its clinical course	2016 WHO classification^18^
Systemic EBV+ T-cell lymphoma of childhood	Name changed from lymphoproliferative disorder to lymphoma owing to its fulminant clinical course and the desire to clearly distinguish it from chronic active EBV infection	

The EBV-associated T- and NK-cell lymphoproliferative diseases include EBV-associated hemophagocytic lymphohistiocytosis (HLH); CAEBV of T- and NK-cell type; systemic EBV+ T-cell lymphoma of childhood; aggressive NK-cell leukemia; extranodal NK/T-cell lymphoma, nasal type; and primary EBV+ nodal NK/T-cell lymphoma (Table 4)^19,20^. Primary EBV+ nodal NK/T-cell lymphoma is a new provisional entity defined as peripheral T-cell lymphoma with primary nodal presentation without nasal involvement^20^. Although splenomegaly and hepatic involvement can occur in 73% and 60% of the patients, skin and gastrointestinal involvement are rare^21^. EBV-associated HLH is a life-threatening inflammatory disease with symptoms including high fever, cytopenia, hypofibrinogenemia, elevated serum transaminases, hyperbilirubinemia, prolonged prothrombin time, and hyponatremia; it may occur as a primary condition or secondary to other conditions^19^. Secondary EBV-associated HLH may accompany CAEBV of T- and NK-cell type. HVLPD is classified as a cutaneous form of CAEBV of T- and NK-cell type. CAEBV of T- and NK- cell types has a broad range of clinical manifestations from indolent cutaneous form to a more severe systemic form^20^. Aggressive NK-cell leukemia is a fulminant disease characterized by systemic neoplastic proliferation of NK-cells^19^. It occurs in young to middle-aged adults. Extranodal NK/T-cell lymphoma, nasal type, is EBV+ aggressive lymphoma and occurs mainly in middle-aged adults. It occurs in 70–80% of all cases and primarily involves the nasal and nasopharyngeal region. Skin lesion presents as nodular ulcerative lesions, and gastrointestinal tract, testis and mucous membrane are involved^19^. Systemic EBV+ T-cell lymphoma of childhood is a more rapidly progressive and fatal disease, characterized by fever, hepatosplenomegaly, liver failure, lymphadenopathy, HLH, and pancytopenia^1,20^. However, as some features overlap among EBV-associated T- and NK-cell lymphoproliferative diseases, the distinction of diseases is sometimes challenging.

**Table 4:** Classification of EBV-associated T- and NK-cell lymphoproliferative diseases^19,20^.

Disease
Aggressive NK-cell leukemia
Chronic active EBV infection (CAEBV) of T- and NK-cell type
Cutaneous CAEBV of T- and NK-cell type
Hydroa vacciniforme-like lymphoproliferative disease
Severe mosquito bite allergy
Systemic CAEBV of T- and NK-cell type
EBV-associated hemophagocytic lymphohistiocytosis
Extranodal NK/T-cell lymphoma, nasal type
Primary EBV+ nodal NK/T-cell lymphoma^a^
Systemic EBV+ T-cell lymphoma of childhood

^a^A provisional entity.

HVLPD occurs in the presence of chronic EBV infection and presents characteristic sun-related skin eruptions. Although patients with HVLPD can show a favorable prognosis, some patients may have significantly serious outcomes, including progression to systemic lymphoma. These cases with extracutaneous involvement of atypical lesional cells are diagnosed as systemic EBV+ T-cell lymphoma of childhood according to the revised 4th WHO classification^1,2^. Although classic HVLPD usually occurs in the pediatric age group, there have been cases of systemic EBV+ T-cell lymphoma in adults with HVLPD^3,5,22^.

The prognostic factors of progression from HVLPD to systemic lymphoma have not been definitely established. Factors such as high serum EBV DNA loads, Latin American descent, systemic symptoms, adult onset, and chemotherapy were suggested to be associated with a more aggressive disease course^3,10,19,20^. In our database, among 19 adult cases, 11 (57.9%) were associated with systemic lymphoma, and this proportion was higher than that observed among childhood cases. Among these cases, seven progressed to systemic EBV+ T-cell lymphoma; three to extranodal NK/T-cell lymphoma, nasal type; and one to EBV+ Hodgkin lymphoma. Therefore, considering the higher possibility of adverse outcomes, adult-onset cases may also need careful monitoring as in children cases.

There is no established treatment guideline for HVLPD. Stringent sun protection is recommended for all patients using broad-spectrum sunscreens, sun-protecting clothing, and avoidance of sun exposure. Various treatments were tried including hydroxychloroquine, anti-viral drugs (acyclovir and valacyclovir), topical and systemic corticosteroids, and chemotherapy. For systemic HVLPD, although corticosteroids or thalidomide provided temporary improvements, the only curative treatment is hematopoietic stem cell transplantation^23^. A recent report suggested that chemotherapy should not be considered as the first-line treatment as it was associated with poor prognosis in patients with HVLPD^3^. However, chemotherapy may be an effective method to reduce systemic disease burden in an effort to enhance the curative potential of allogeneic bone marrow transplantation, with or without the reduction of EBV viral load^24^. Therefore, its use should be prudently considered in selected cases. Further investigations on which patients can benefit from chemotherapy are warranted.

There are some limitations. Although this study included one of the large cases with three decades data in SNUH, the sample size is small as HVLPD is an extremely rare disease. In addition, a detailed molecular analysis was not performed due to the retrospective study design. Lastly, it was challenging to distinguish between HVLPD and extranodal NK/T-cell lymphoma, nasal type, and primary EBV+ nodal T/NK-cell lymphoma with extranodal involvement as some features are overlapped. However, the diagnosis was made based on clinical and pathologic findings by an experienced dermatologist, pathologist, and oncologist.

In summary, we described 26 cases of HVLPD encountered at our institution during the last three decades. HVLPD seems to have various disease courses. Fourteen patients (53.8%) in the present study had systemic T-cell lymphoma, NK-T cell lymphoma/leukemia, and EBV+ Hodgkin lymphoma. Lymphoma progression and mortality can occur not only in childhood but also in adulthood.

## Methods

We retrospectively reviewed cases of HVLPD diagnosed based on clinical and histopathologic features between 1988 and 2019 in the Department of Dermatology of SNUH. Patients who both gave and signed an informed consent to perform a skin biopsy were recruited (In cases of patients under 18 years of age, signed informed consent were given by parents and/or legal guardians). HVLPD was defined according to the revised 4th WHO classification of hematopoietic and lymphoid tumors^1^. Clinical and laboratory data at the time of diagnosis were obtained from medical records.

Histopathologic features, immunohistochemical findings, EBER by in situ hybridization, serum EBV loads, T-cell clonality test using multiplex-PCR for the T-cell receptor γ gene, and results of radiologic imaging studies were reviewed. Photoprovocation tests UVA. A 5 × 5 cm area on the buttock or upper arm was irradiated 3 or 5 times at 24-h intervals (15–20 J/cm^2^)^9^. This study was approved by the institutional review board of SNUH (approval no. 1908-019-1053). All methods were carried out in accordance with relevant guidelines and regulations.

Statistical analyses were performed using SPSS 25 (SPSS Inc., IL, USA). Pearson’s chi-square test was performed to compare proportions, except when the expected values in the SPSS cells were < 5, in which case Fisher’s exact test was used. Student’s t-test was used to compare mean disease durations.
